# Barriers and Strategies of Cationic Liposomes for Cancer Gene Therapy

**DOI:** 10.1016/j.omtm.2020.07.015

**Published:** 2020-07-31

**Authors:** Chunyan Liu, Ligang Zhang, Wenhui Zhu, Raoqing Guo, Huamin Sun, Xi Chen, Ning Deng

**Affiliations:** 1 Guangdong Province Engineering Research Center for Antibody Drug and Immunoassay, Department of Biology, Jinan University, Guangzhou 510632, China

**Keywords:** cationic liposomes, lipoplexes, gene transfer, extracellular barriers, intracellular barriers, lipid compositions, cancer gene therapy

## Abstract

Cationic liposomes (CLs) have been regarded as the most promising gene delivery vectors for decades with the advantages of excellent biodegradability, biocompatibility, and high nucleic acid encapsulation efficiency. However, the clinical use of CLs in cancer gene therapy is limited because of many uncertain factors *in vivo*. Extracellular barriers such as opsonization, rapid clearance by the reticuloendothelial system and poor tumor penetration, and intracellular barriers, including endosomal/lysosomal entrapped network and restricted diffusion to the nucleus, make CLs not the ideal vector for transferring extrinsic genes in the body. However, the obstacles in achieving productive therapeutic effects of nucleic acids can be addressed by tailoring the properties of CLs, which are influenced by lipid compositions and surface modification. This review focuses on the physiological barriers of CLs against cancer gene therapy and the effects of lipid compositions on governing transfection efficiency, and it briefly discusses the impacts of particle size, membrane charge density, and surface modification on the fate of CLs *in vivo*, which may provide guidance for their preclinical studies.

## Graphical Abstract

**Figure:**
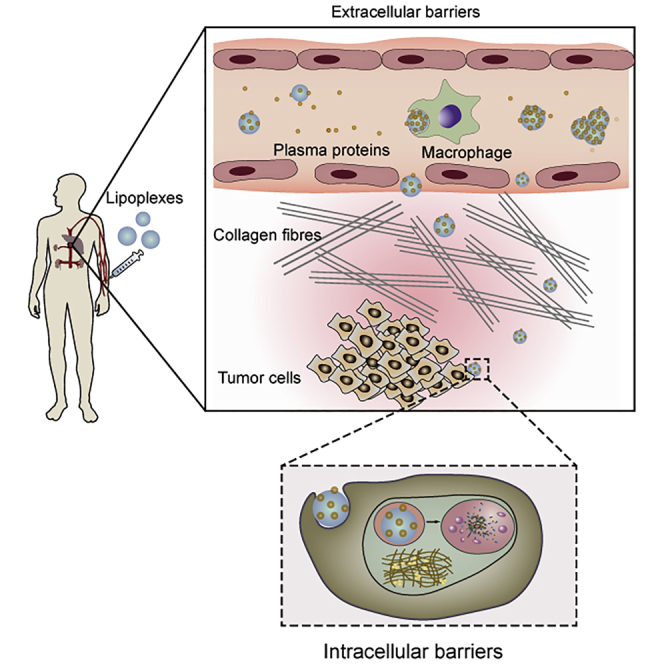

## Teaser

The concept of lipoplexes applied to cancer gene therapy has been popular for decades; however, no such medicine has been marketed. Deng and colleagues highlight the physiological barriers against the clinical success of the cationic liposomal gene delivery system in cancer therapy. They suggest that the rational design of clinically applicable lipoplexes requires designers to pay close attention to the barriers and the impact of physicochemical properties of cationic liposomes on the therapeutic outcome.

## Main Text

Cationic liposomes (CLs) are near-spherical vesicles with a positive charge and are dominantly composed of cationic lipids that effectively condense nucleic acids^1^, ^2^, ^3^, ^4^ such as plasmid DNA (pDNA), messenger RNA (mRNA), or small interfering RNA (siRNA) via electrostatic interactions^5^ to form complexes called “lipoplexes.” Based on the fact that genes have been revealed to be involved in the development of cancer,^6^^,^^7^ CLs have been extensively studied in cancer gene therapy and have shown potential clinical success in different tumors (Table 1). In general, the selection of CLs as gene carriers is attributed to their permanent positive charge, which promotes their stronger interaction with negatively charged substances such as nucleic acid, cell membrane, and endosomal membrane. In addition, CLs can be adjusted to obtain appropriate physical and chemical properties, such as size and tumor targeting, so as to obtain good pharmacokinetic properties *in vivo*. However, despite the good performance showed in *in vitro* studies, cationic lipid-based nucleic acid therapies in clinical trials are less than satisfactory because of the complicated biological environment.^8^ Most clinical trials of cationic liposomal delivery systems for cancer gene therapy have been approved (Table 2), but most are still in phase I or even discontinued.

**Table 1:** The Preclinical Studies of Cancer Gene Therapy Involving Lipoplexes

Name	Drug	Carrier Components	Diameter (nm)	Disease	References
T7-LPC	EGFR siRNA	DOTAP/DOPE/Chol, 1:1:1 n/n, 5% T7-PEG-DSPE	83	glioma	^185^
PEGylated DC-Chol/DOPE cationic liposomes	KSP siRNA	DC-Chol/DOPE/mPEG-2000-DSPE, 195:195:1 n/n	102	ovarian cancer	^47^
DLPP	IL-22BP mRNA	DOTAP/Chol, 1:1 n/n	157	colon cancer	^186^
pegSA lipoplexes	BMP-9 pDNA	fSA/HSPC/DOPE/DSPE-mPEG-2000/Chol	<100	osteoporosis	^187^
FRα-targeted liposomes	matrix protein pDNA	DOTAP/Chol/mPEG-suc-Chol/F-PEG-suc-Chol, 50:45:4.75:0.25, n/n	200	ovarian cancer	^188^
HA lipoplexes	CD44 siRNA	HA-DOPE/DE, 1:1 w/w	230	lung cancer	^189^
2X3-DOPE/FC liposomes	MDR1 siRNA	2X3/DOPE, 1:2 n/n	60	squamous carcinoma	^171^
HA-P-LP	shRNA mRIP3-pDNA	DOTAP/DOPE/PEG-DSPE/Chol	290	colon cancer	^158^
CL-siSOX2	SOX2 siRNA	DOTAP/DPPC/DSPE-mPEG-2000, 2:3:2 n/n	93	lung cancer	^190^
DACC	CD31 siRNA	AtuFECT01/Chol/mPEG-2000-DSPE, 70:29:1 n/n	~70	lung cancer	^191^
Liposome-siRNA nanocomplex	STAT3 siRNA, curcumin	DOTAP/DOPE/sodium cholate/C6 ceramide, 50:30:10:10 w/w	157	skin cancer	^192^

EGFR, epidermal growth factor receptor; Chol, cholesterol; DSPE, 1,2-distearoyl-*sn*-glycero-3-phosphorylethanolamine; KSP, kinesin spindle protein; IL-22BP, interleukin-22 binding protein; pegSA, PEGylated stearyl amine; BMP-9, bone morphogenetic protein-9; FRα, folate receptor α; suc, succinyl; HA, hyaluronic acid; DE, [2-(2-3-didodecyloxypropyl)hydroxyethyl]ammonium bromide; 2X3, 1,26-bis(cholest-5-en-3β-yloxycarbonylamino)-7,11,16,20-tetraazahexacosan tetrahydrochloride; FC liposomes, folate-containing lipoconjugate liposomes; SOX2, SRY HMG-box 2; AtuFECT01, β-l-arginyl-2,3-l-diaminopropionic acid-*N*-palmityl-*N*-oleyl-amide trihydrochloride; DLPP, DOTAP liposome-protamine complex; CD31, platelet endothelial cell adhesion molecule-1; STAT3, signal transducer and activator of transcription 3; n/n, molar ratio; w/w, weight ratio.

**Table 2:** Cationic Liposomal Delivery System for Cancer Gene Therapy in Clinical Trials

Name	Drug	Carrier Components	Administration Route	Condition	ClinicalTrials.gov Identifier (Phase)	Sponsor and/or Affiliations	First Posted
Atu027	PKN3 siRNA	AtuFECT01-DPhyPE/DSPE-PEG-2000	i.v.	pancreatic cancer	NCT01808638 (I/II)	Silence Therapeutics	2013
SGT-94	RB94 pDNA	DOTAP/DOPE	i.v.	solid tumors	NCT01517464 (I)	Synergene Therapeutics	2012
SGT-53	HWTp53 pDNA, PD1 antibody	DOTAP/DOPE	i.v.	glioblastoma, solid tumors, pancreatic cancer	NCT02340156 (II)	Synergene Therapeutics	2015
					NCT02340117 (II)		
Lipo-MERIT	(NY-ESO-1, MAGE-A3, tyrosinase and TPTE) RNAs	DOTMA/DOPE	i.v.	stage IIIB–IV melanoma	NCT02410733 (I)	Biopharmaceutical New Technologies	2015
Tusc2-nanoparticles	Tusc2 pDNA	DOTAP/Chol	i.v.	lung cancer	NCT01455389 (I/II)	MD Anderson Cancer Center	2011
Liposomal-DNA complexes	interleukin-2 pDNA	DOTMA/Chol	i.t.	head and neck cancer	NCT00006033 (II)	H. Lee Moffitt Cancer Center and Research Institute	2004
DC-Chol liposomes	EGFR antisense DNA	DC-Chol	i.t.	head and neck cancer	NCT00009841 (I)	University of Pittsburgh	2004
pbi-shRNA STMN1 lipoplexes	bi-shRNA-stathmin 1 pDNA	DOTAP/Chol	i.t.	solid tumors	NCT01505153 (I)	Gradalis	2012
IGTM-101	(HSTK, cIFNβ, hIL-2, hGM-CSF) pDNA	DMRIE/DOPE	intratumoral/peritumoral injection	melanoma	NCT03338777 (I) terminated^a^	Hospital Italiano de Buenos Aires	2017

Source: https://clinicaltrials.gov. PKN3, protein kinase N3; DPhyPE, 1,2-diphytanoyl-*sn*-glycero-3-phosphoethanolamine; RB94, an N-terminal truncated retinoblastoma protein; HWTp53, human wild-type tumor protein 53; PD1, programmed cell death-1; NY-ESO-1, New York esophageal squamous cell carcinoma 1; MAGE-A3 melanoma antigen family A3; TPTE, transmembrane phosphatase with tensin homology; Tusc2, tumor suppressor candidate 2; STMN1, stathmin 1; DOTMA, *N*-[1-(2,3-dioleyloxy)propyl]-*N*,*N*,*N*-trimethylammonium chloride; HSTK, herpes simplex thymidine kinase; cIFNβ, canine interferon β; hIL-2, human interleukin-2; hGM-CSF, human granulocyte-macrophage colony-stimulating factor; DMRIE, 1,2-dimyristyl oxypropyl-3-dimethyl-hydroxyethylammonium bromide; i.v., intravenous; i.t., intratumoral.

a Failure to achieve primary objective, terminated at 2020.

The efficiency of CLs in gene delivery is affected by many factors *in vivo* and may not closely correspond to the studies *in vitro*.^9^^,^^10^ First, CLs can elicit opsonization after intravenous administration.^11^ CLs pick up plasma protein such as serum albumin, complements, immunoglobulins (Igs), and apolipoproteins to form a corona layer on their surface, providing them with a totally new biological identity that will dramatically alter their fates *in vivo*.^12^^,^^13^ Meanwhile, the reticuloendothelial system (RES) will first play the role of eliminating aliens to remove CLs from the blood circulation, and the inappropriate size and excessive positive charge of CLs accelerate this process.^10^^,^^14^^,^^15^ In addition, even if they manage to escape the RES trap and extravasate from vasculatures to the tumor tissues, particle characteristics determine whether they can successfully penetrate from the complex tumor environment into tumor cells, followed by effective ingestion via efficient internalization. Besides, endosomal/lysosomal entrapment is another obstacle for CLs to deliver genes to cytoplasm,^16^, ^17^, ^18^ which is greatly affected by the fusogenic capability of CLs.^19^ Furthermore, the reticular cytoskeletal network and the macromolecular crowding in the cytoplasm are not conducive to the transport of lipoplexes or DNA to the nucleus.^20^^,^^21^ Therefore, even though CLs have been considered to be the most promising nanocarriers for cancer gene therapy, gene delivery to target sites is bumpy, because CLs, as invaders, face a series of events such as opsonization, rapid clearance by the RES, poor tumor penetration, cellular uptake, and lysosomal degradation, resulting in therapeutic failure in the body.

The intracorporal fates of CLs are not only affected by the physiological environment but also by the physiochemical properties of CLs, while the latter are primarily determined by lipid compositions.^8^^,^^22^, ^23^, ^24^ CLs are usually composed of the cationic lipids and neutral helper lipids such as cholesterol, dioleoyl phosphatidylethanolamine (DOPE), and polyethylene glycol (PEG)-lipid, among others. Notably, the molecular structure of cationic lipid and the ability of neutral helper lipid to trigger fusion are of great importance to govern transfection efficiency (TE).^9^^,^^25^, ^26^, ^27^ It has been reported that TE is highly correlated with nanoparticle size and surface charge, both of which are affected by lipid composition.^28^^,^^29^ Additionally, the active targeting and appropriate characteristics of CLs can also be obtained by simply tailoring the lipid compositions, which are crucial to the outperformance of CLs in the body. Therefore, the rational design of CLs requires a full understanding of the relationship between lipid composition and therapeutic outcome.

The clinical research of lipoplex-based therapeutic agents in cancer treatment has made slow progress in the last two decades due to the complicated environment and poor therapeutic outcome in the human body. Therefore, understanding the factors affecting CL-based gene therapy is crucial for the design of efficient and clinically applicable gene delivery vectors.

### The Extracellular Barriers

#### The Protein Corona Alters the Fate of CLs *In Vivo*

**Figure 1:**
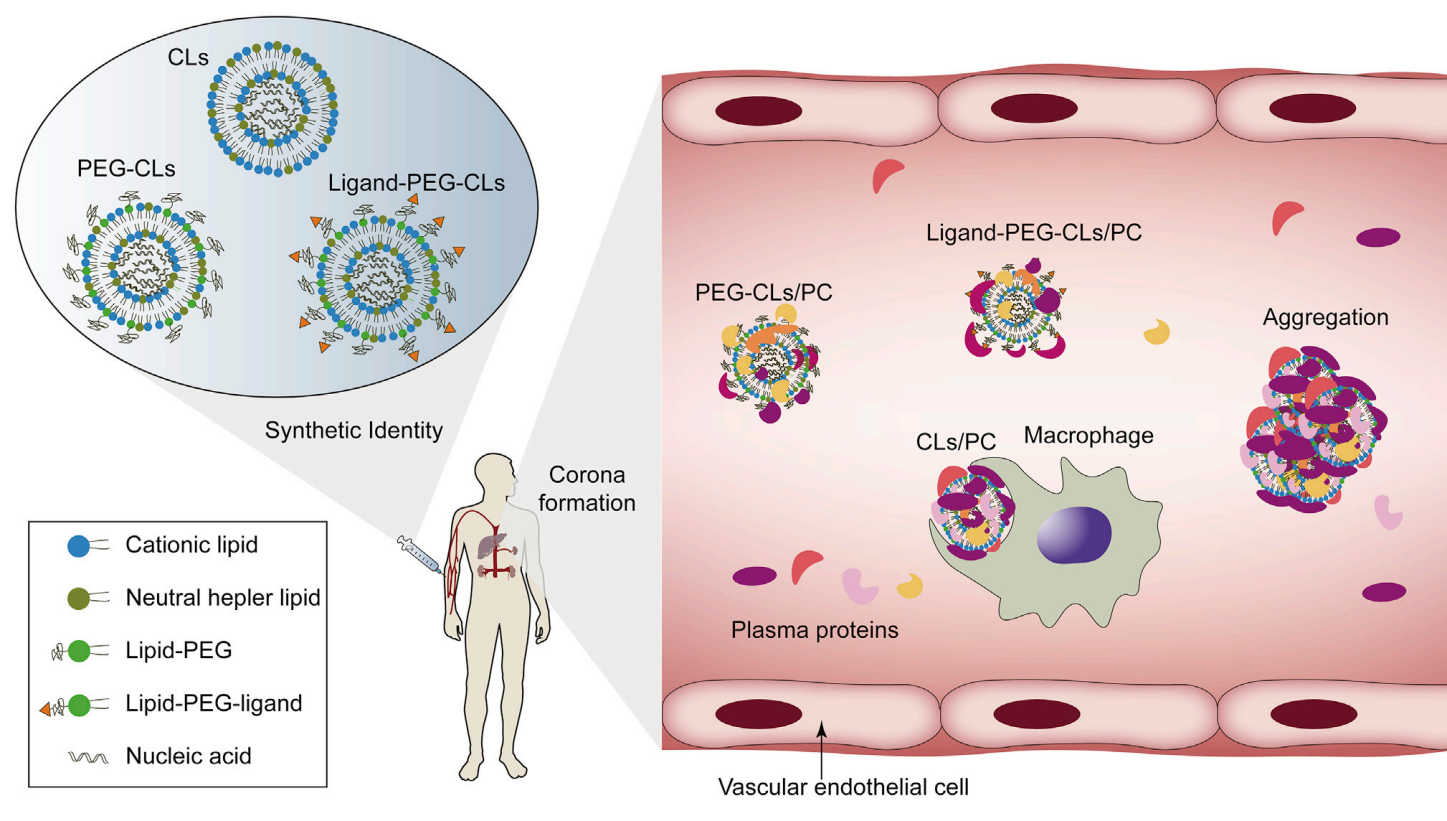
Schematic Representation of the Effects of PC on CLs The decoration of PC obscures the surface charge and targeting ligands of CLs, increases their size, and leads to aggregation and off-target. Most of them are quickly cleared by macrophages. PEG-CLs reduce the adsorption of proteins to some extent.

Once administered intravenously, the plasma proteins will adsorb to CLs or lipoplexes and eventually form a so called “protein corona” (PC) at their surface that can specifically bind to phagocytes (Figure 1).^30^^,^^31^ The absorption of complement protein activates the complement system,^32^ while Ig promotes the opsonization of CLs,^33^ with both resulting in rapid clearance of CLs. However, based on the immunogenicity of CLs, some researchers have exploited this property of liposomes to target immune cells such as tumor-associated macrophages and dendritic cells.^34^^,^^35^ In antitumor immunotherapy or vaccination, CLs can also serve as an attractive adjuvant^36^ in addition to its carrier function, because CLs themselves can elicit the release of cytokine and the activation of natural killer (NK) cells, resulting in a greatly enhanced pro-inflammatory response around the tumor tissue.^37^^,^^38^

Despite this, the impacts of PC on CLs are sometimes fatal, such as aggregation, charge neutrality, and size enlargement, leading to their instability and drug release before cell uptake.^39^^,^^40^ Meanwhile, the active moieties of ligand-modified CLs may also be shielded after opsonization.^41^ Several recent findings have confirmed that the decoration of PC on nanocarriers could alter their internalization mechanism and hence intracellular trafficking fate.^12^^,^^39^^,^^42^ All of these changes will produce different clinical effects than expected, which is one of the reasons for the little clinical progress of nanocarriers in recent decades. Importantly, however, note that the isolated serum or plasma is not completely consistent with the actual physiological environment, which means that *in vitro* experiments on liposomes cannot be fully used to predict their behavioral changes *in vivo*.^43^

Many efforts have been made to avoid the negative effects of corona, among which PEGylation^44^^,^^45^ is more effective and commonly used. Attaching PEG chains creates a shielding layer at the surface of CLs and hence the formation of steric barriers and the reduction of exposed charges,^46^ resulting in less formation of PC and extension of circulation time in the body.^47^ However, the stealth effect of PEGylated CLs is not conducive to cellular uptake and endosomal escape.^48^^,^^49^ By adjusting the PEG chain length and PEG density, the effects of anti-opsonization and inhibition of lipid-plasma membrane interactions caused by “PEG dilemma” can be balanced.^49^^,^^50^ Alternatively, the addition of a targeting ligand and a pH-sensitive fusogenic peptide,^51^ as well as introducing cleavable PEG-lipids to the liposomal formulation, not only helps PEGylated CLs^52^^,^^53^ to maintain their stealth role in blood circulation, but promotes penetration into target cells and subsequent endosomal escape. However, similar immune response problems can occur with repeated intravenous administration of PEGylated liposomes. A brief report by McSweeney et al.^54^ disclosed that PEG-modified therapeutics can induce specific PEG-binding antibodies, leading to rapid elimination of drugs and a significantly increased risk of serious adverse events.

The protein abundance of corona is affected by particle size and charge, which vary with lipid compositions.^41^^,^^55^^,^^56^ Ren et al.^57^ investigated whether liposomes with a slight negative charge might enhance their blood circulation time due to reduced combinations with plasma proteins and blood cells compared to CLs. However, after extensive experiments on the effects of lipid compositions on corona protein components, it has been discovered that not all of the formation of PC has an adverse effect on CLs. The adsorbed proteins can be functionally divided into opsonins and dysopsonins, while the later may give CLs a desirable biological identity that prolongs their blood circulation time or enhances accumulation in tumors.^58^ Caracciolo et al.^59^ found that 1,2-dioleoyl-3-trimethylammonium-propane (DOTAP)-rich liposomes preferred vitronectin, whose receptor is highly expressed in tumor cells, while dimethylaminoethane-carbamoyl (DC)-cholesterol promoted the binding of Ig and complement proteins. Nevertheless, substitution of the cationic lipid DOTAP with neutral lipids such as DOPE decreases the net charge of the lipid vesicle, making it less attractive for vitronectin, fibrinogen, and other negatively charged proteins,^60^ but more attractive for dysopsonins such as apolipoproteins and serum albumin, which preferentially adhere to the DOPE molecules.^61^^,^^62^ In recent years, protein adsorption has been used to induce the formation of functional PCs to target tumor cells.^11^^,^^56^^,^^63^^,^^64^ These important findings suggest that researchers should take PC into account and design rationally “targeted” CLs (simply via optimizing the lipid compositions) so as to take advantage of the physiological environment as much as possible.^11^^,^^65^

#### The Insufficient EPR Effect and Poor Tumor Penetration

**Figure 2:**
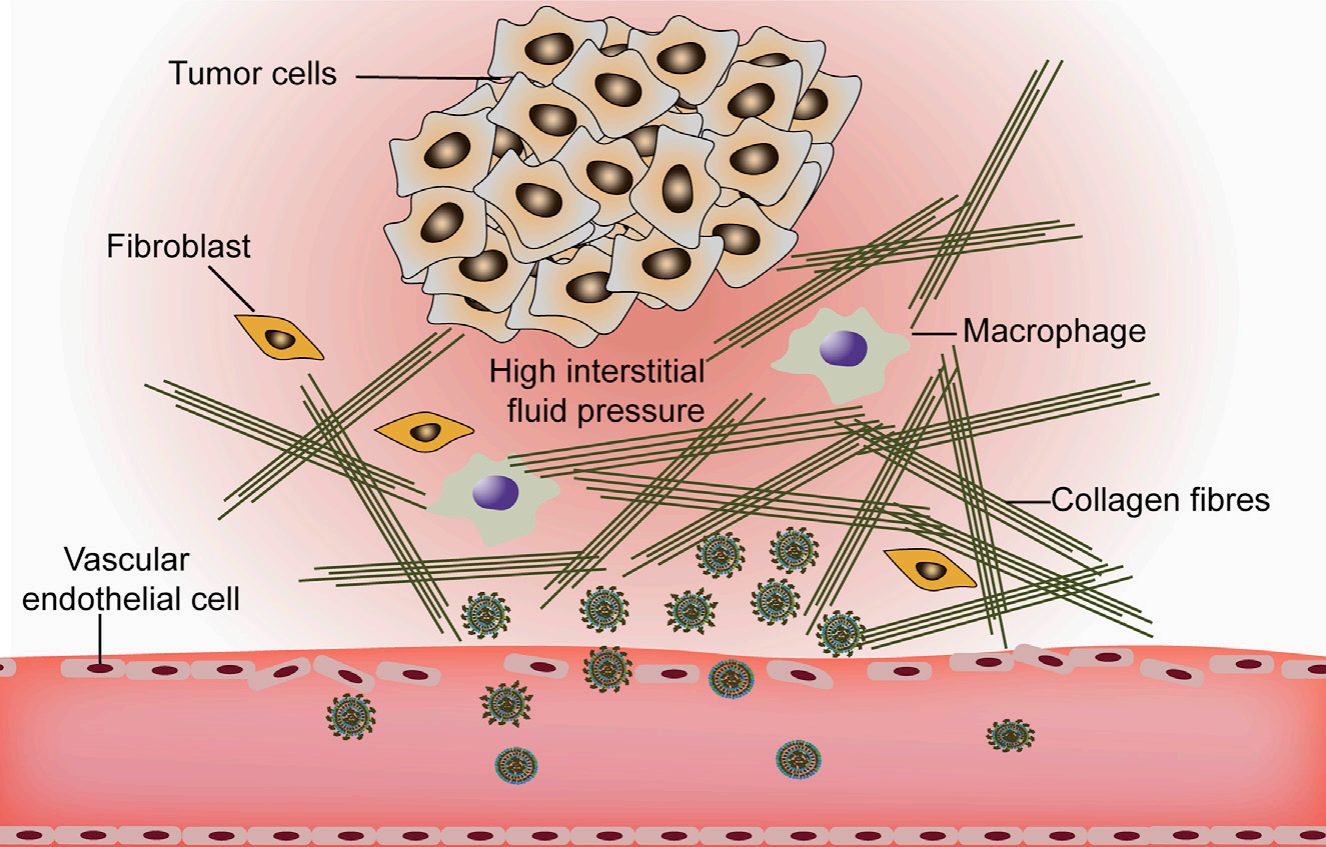
Illustration of Tumor Microenvironment Vascular abnormalities and lack of lymphatic drainage allow CLs to accumulate in tumor, but dense extracellular matrix and increased interstitial fluid pressure prevent the particles from penetrating deeper into the tumor.

The advantage of CLs in the treatment of cancer, as with other nanomedicine, is that they can passively target tumor tissues because of the enhanced permeability and retention (EPR) effect, which is caused by leakiness of the neovasculature and inefficient lymphatic drainage in tumors.^66^^,^^67^ Rapid tumor growth leads to angiogenesis with pore diameter ranging from 100 to 800 nm,^68^ which allows molecules or particles larger than 40 kDa to extravasate from blood vessels^69^ and accumulate in tumor tissues, providing a huge opportunity for preferential tumor accumulation of nanomedicines. Therefore, there is an understanding that by simply optimizing the particle size (ranging from 100 to 200 nm, with 100 nm being commonly used^70^), effective passive tumor targeting can be designed based on the EPR effect. The EPR effect is not limited to tumors, as Ren et al.^57^ discovered that the optimal particle size for liposomes in rheumatoid arthritis targeting therapy was 100 nm, while larger (200 and 350 nm) and smaller (70 nm) liposomes had shorter circulation times due to their easier recognition by the RES and filtration by kidney, respectively. However, the cutoff sizes of capillary pores or optimal sizes for EPR-based drug delivery system are in conflict in several articles,^68^^,^^71^, ^72^, ^73^, ^74^, ^75^, ^76^ and the invalid delivery of nanocarriers into tumors exhibits an insufficient EPR effect due to the heterogeneity of tumor.^67^^,^^77^ In fact, the EPR effect is a phenomenon that is unique to cancer patients and relies on blood vessels with defective architecture.^78^ More specifically, the gaps between endothelial cells in tumor blood vessels and the vascular density as well as the blood supply are key determinants for EPR-mediated tumor targeting.^79^ The convection of lipoplexes is blocked by the increased interstitial fluid pressure (IFP), which is induced by abnormal vasculature, ineffective lymphatic drainage, interstitial fibrosis, and stromal matrix contraction (Figure 2).^80^, ^81^, ^82^ Differences in tumor size and vascular density of different stages, accompanied by a special tumor microenvironment, such as an abnormal tumor vascular system, dense extracellular matrix (ECM), and high IFP, make the EPR effect alone unsatisfactory for all solid tumors.^69^^,^^83^^,^^84^ Furthermore, abnormal ECM, together with the increased IFP, makes lipoplexes almost motionless in the periphery of the tumor.^85^

Particle size is the most important factor that influences the elimination, tumor penetration, and retention of nanomedicine due to the inconsistent cutoff sizes in different sites, including kidney (<10–15 nm),^86^ liver (50–180 nm),^8^ leaky vasculature (>200 nm), and ECM (<20 nm),^87^ among others. Particles that are too small (<10 nm) or slightly larger (>50 nm) are not suitable for the retention and penetration in tumors;^88^^,^^89^ therefore, designing nanocarriers with intelligent tunable sizes is the most controllable way to balance the size-related accumulation and penetration abilities.^90^ For example, near-infrared light triggered size-tunable liposomes not only extended the blood circulation time, but also promoted their penetration into deep tumor after the size decreased from large (~162 nm) to small (~8.6 nm).^91^ Importantly, note that the actual size of effective penetration in a tumor varies with different delivery systems, tumor types, and individuals.^84^ More uplifting news is that transcytosis of nanomedicine with a positive charge and certain ligand modification has been shown to mediate the active intra-tumor penetration without the need to overcome the hindrance of ECM.^92^ Transcellular transport occurs in any nanoparticles modified with peptides containing C-terminal arginine that bind to neuropilin-1 or neuropilin-2 on the tumor plasma membrane.^84^ This is very exciting news for scientists to design a highly effective tumor-permeable gene delivery system, because it allows nanomedicines to target the vascular endothelial cells and trigger adsorption or receptor-mediated endocytosis for exocytosis to the adjacent cell on the other side without size limitation.^85^

Another promising but more traditional strategy for enhancing tumor penetration is a prior administration of liposomes encapsulating chemotherapy drugs to deplete the dense ECM, and then followed by liposomes loaded with genetic drugs.^93^ For example, Chen et al.^94^ successfully delivered drug-loaded liposomes into the stromal abundant pancreatic ductal adenocarcinoma due to the stromal depletion that was achieved by pretreatment with nitric oxide donor *S*-nitroso-*N*-acetylpenicillamine-loaded liposomes.

### Intracellular Barriers

#### Cellular Entry

**Figure 3:**
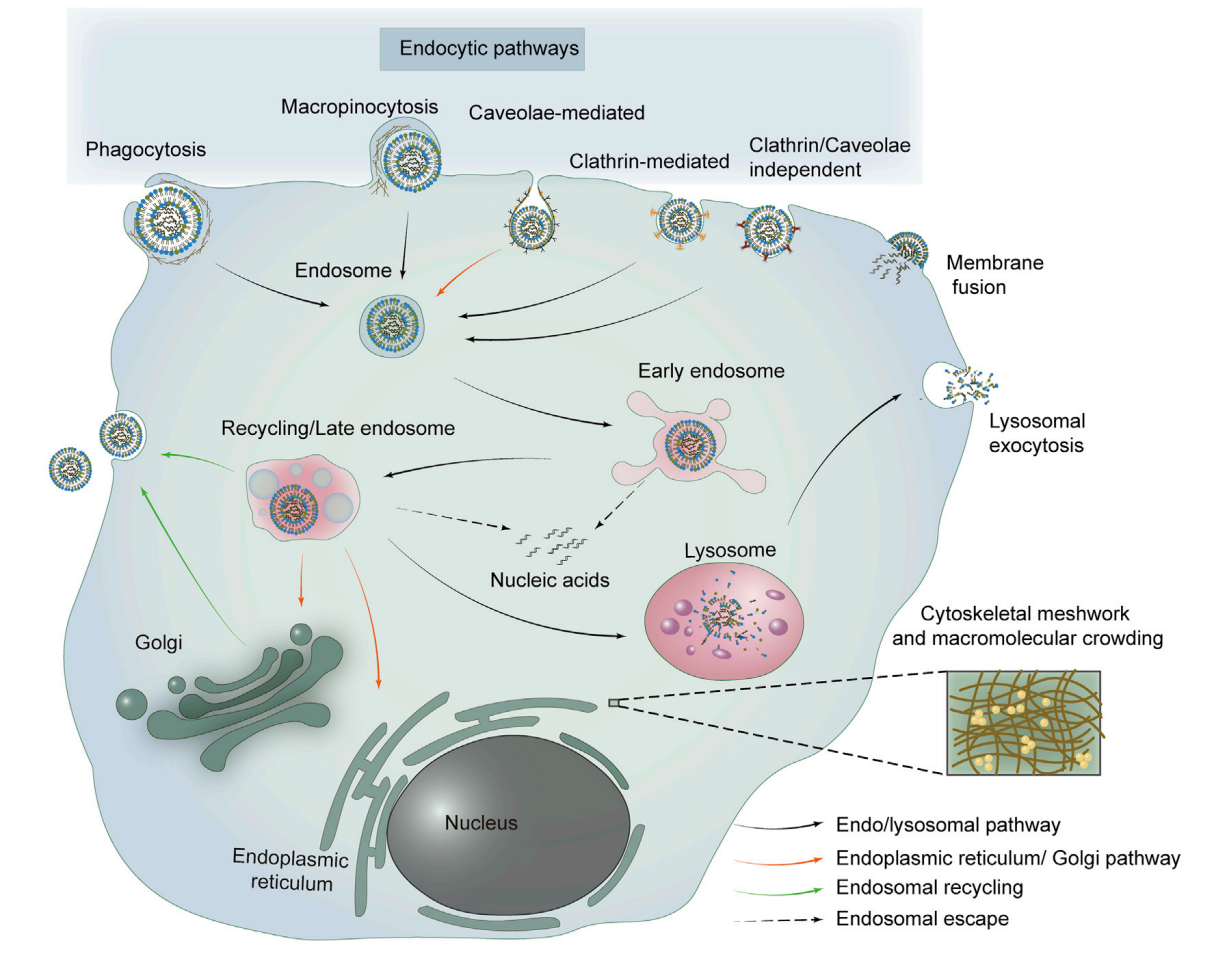
Schematic Diagram of Lipoplexes Entering and Trafficking Within Cells: Entry Pathways and Intracellular Barriers Lipoplexes enter into cells via endocytosis or membrane fusion. Endocytic pathways include clathrin-mediated endocytosis, caveolae-mediated endocytosis, phagocytosis, micropinocytosis, and clathrin/caveolae-independent endocytosis. CLs must escape the endosomal entrapment before being transported to the lysosome except for membrane fusion and caveolae-mediated endocytosis. Furthermore, the mesh-like cytoskeletal network and macromolecular crowding in cytoplasm impede the diffusion of lipoplexes to the nucleus.

When CLs surf onto the surface of the cell membrane, cellular uptake may occur through endocytosis or membrane fusion (Figure 3). In contrast, membrane fusion occurs almost exclusively in fusogenic CLs,^95^^,^^96^ which directly translocate their cargo into the cytoplasm,^97^^,^^98^ resulting in efficient drug delivery to the cytoplasm without endosomal entrapment and metabolic degradation.^99^

CLs that exist in a lamellar phase are known to be internalized by cells mainly through endocytosis,^100^ which can be divided into clathrin-mediated endocytosis (CME), caveolae-mediated endocytosis (CavME), phagocytosis, macropinocytosis, and clathrin/caveolae-independent endocytosis.^101^, ^102^, ^103^ Among the several endocytic pathways, CME, CavME, and macropinocytosis are more common in the cellular uptake of lipoplexes in ordinary cells (normal or cancerous cells of various tissues other than phagocytes). In particular, phagocytosis, which professional phagocytic cells use to internalize micro-size particles (such as dead cells or pathogens), is closely related to the intracorporal fate of CLs after opsonization in blood, as mentioned previously. Phagocytic cells, such as macrophages, neutrophils, and dendritic cells, recognize CLs by binding the specific composition of PC and, once ingested, the phagocytes digest them completely through lysosomal degradation.^32^ For instance, Erni et al.^104^ tried to deliver pDNA to phagocytic professional antigen-presenting cells by cationic solid lipid microparticles (cSLMs) *in vitro*. However, despite the efficient phagocytosis of cSLMs, TE was not detected in macrophage cell lines but in non-phagocytic cells.

There is a growing awareness that endocytic pathways are closely related to the intracellular fate of cargo and the ease of drug release,^101^^,^^105^ which involve lysosomal degradation. Lysosomes are present in all mammalian cells except mature erythrocytes, but they are most abundant in phagocytes (macrophages and neutrophils)^106^ compared with other cell types. Significantly, it has been reported that tumor cells have an increased content of lysosomes due to their rapid proliferation and metabolization,^107^ which generally leads to drug resistance or inefficacy, especially for weak-base chemotherapeutic agents.^108^ Lysosomal activity, a wide range of biological process involved in digestive function and host defense, relates to the degradation of substances from endocytosis or autophagy.^109^ Cell type^110^^,^^111^ and physicochemical properties of CLs, including size, charge, lipid composition, surface modification, are important factors in endocytosis.^56^^,^^101^ When considering the effect of particle characteristics on endocytosis, the most striking factor is usually the particle size, as clathrin-coated pit and caveolae are size-limited invaginations.^90^^,^^101^ However, the exact size of different endocytic pathways needed remains to be determined. For example, Inoh et al.^112^ investigated the effects of lipoplex sizes on the endocytosis pathway in bone marrow-derived dendritic cells, and they surprisingly found that CavME preferred to internalize smaller-sized lipoplexes (around 270 nm), while CME and macropinocytosis preferred to take up the larger ones (around 500 nm). However, the results of Bae et al.^113^ showed that cholesterol ester liposomes and DOTAP liposomes of different sizes were all internalized in COS-7 cells mainly via the CME pathway. Accordingly, the selection of liposome size for endocytosis does not seem obvious. As research continues, size-dependent internalization is not widely accepted, because even monodispersed liposomes can be internalized simultaneously by the same cell in multiple endocytic pathways.^114^ Kim et al.^115^ observed that particle size and charge had little effect on TE throughout all used cell lines, and it was most dependent on cell lines, and then the lipid compositions. In other words, it is apt to be cell line-dependent rather than the particle size or charge that influences the cellular endocytosis.

#### Endosomal Escape and Drug Release

Regardless of the route of endocytosis, CLs or lipoplexes are enveloped by endosomes and undergo maturation with the gradual acidification of the lumen, i.e., from early endosomes to recycling/multivesicular late endosomes, during which the wrapped CLs are sorted and directed to their destination (i.e., the plasma membrane, endoplasmic reticulum, *trans*-Golgi network, or lysosomes).^102^ However, not every endocytic pathway leads to a productive release of cargo, and the key to their effectiveness of the cytosolic delivery is to avoid the endosomal/lysosomal pathway.^116^^,^^117^ Unfortunately, the late endosomes prefer to transport their preys to lysosomes for further degradation, except CavME, which has been shown to bypass trafficking into lysosomes, resulting in higher TE (Figure 3).^118^^,^^119^ Although endosomal vesicles formed by macropinocytosis are inherently leaky, leading to only a small portion of drug leakage into cytosol without destabilizing the endosomal membrane, they are doomed to destined to the lysosomes by the microtubule network.^111^^,^^120^ Thus, if endocytosing is not via CavME, it is necessary for CLs to disrupt the endosomal membrane quickly so as to release their genetic cargos or incomplete lipoplexes to the cytoplasm before degradation by hydrolases in lysosomes.^121^^,^^122^

Endosomal escape, which relies on membrane fusion, is an indispensable step for lipoplexes trapped in endosomes to acquire satisfactory nucleic acid therapeutic outcome, as it is generally accompanied by drug release.^123^, ^124^, ^125^ The positive charge of the lipoplex attracts the anionic lipids of the endosomal membrane to flip-flop from the outer face to the inner face and diffuse into the lipoplex, forming a neutral ion pair with cationic lipids and resulting in displacement of DNA from the lipoplex as well as endosomal disruption.^124^ During the same time, the nucleic acid can be released into the cytoplasm when the total cationic charge of the lipoplex is completely neutralized by the endosomal membrane.^126^ However, effective DNA dissociation from the carrier is not easy: the thermodynamic stability and membrane charge density (σ_M_, average charge/unit area of membrane) of lipoplexes strongly affect their cargo release.^127^^,^^128^ The thermodynamic stability of lipoplexes is greatly influenced by their phase structure; that is, the lamellar phase is considered to be more thermodynamically stable than the hexagonal phase, which requires lower energy to trigger membrane fusion.^125^ In addition, the lamellar complex needs complete fragmentation to release DNA, while the hexagonal complex can release DNA even if it is not completely disintegrated.^127^ Therefore, an excellent gene delivery vector should be capable of phase transition to meet the requirements of effective drug delivery and drug dissociation. Besides the capability of phase transition, the σ_M_ of lipoplexes is also critical to the endosomal escape and DNA dissociation. Although initial conditions for the escape of lipoplexes from endosome require liposomes with high σ_M_, an excessive positive charge may lead to incomplete dissociation of complexes,^128^ because of the incomplete fusion that occurs only in the external membrane of the lipoplex. Therefore, the positive charge of lipoplexes requires a compromise between endosomal escape and complex dissociation.

#### Migration from Cytoplasm to Nucleus

Endosomal escape and DNA dissociation from lipoplexes are not necessarily a guarantee of successful transfection except for RNA-based cargo; the final obstacle is entry into the nucleus from cytoplasm. The cytoplasm is a mesh-like cytoskeletal network and macromolecular crowding in which only particles with a diameter less than 50 nm can diffuse freely, which inevitably increase the steric hindrance and random collision for the diffusion of lipoplexes or dissociative DNA (Figure 3).^20^^,^^21^^,^^129^ An earlier study by Lukacs et al.^130^ found that only small DNA fragments (<250 bp) diffused rapidly to the nucleus by Brownian motion after microinjection into the cytoplasm, and the movement of the larger counterpart (>2,000 bp) was almost retarded.^129^ Besides size-dependent geometrical constraints, non-specific interaction between nanocarriers and intracellular constituents, such as vesicles, organelles, and internal membranes, is another important factor affecting cytoplasmic diffusion.^20^

Except for cells that are undergoing mitosis or division where their nuclear envelopes are temporarily disassembled,^131^, ^132^, ^133^ nano-size lipoplexes do not appear to be able to freely diffuse into the peripheral nucleus through the cytoskeleton meshwork, let alone into the nucleus through the size-limited nuclear pore complexes.^134^^,^^135^ Apparently, it cannot be true since transfections do work, and there must be other means involving lipoplexes actively trafficking to the nucleus.^132^ Several studies have found that the microtubule network mediates the directed transport of lipoplexes in the cytoplasm to the nucleus, which contributes to the high TE.^131^^,^^136^, ^137^, ^138^ Conversely, Hasegawa et al.^139^ found that the microtubule network actively transported lipoplexes to the lysosomes rather than to the nucleus.^140^ However, Brownian diffusion of lipoplexes was still observed even when the microtubule pathway was disrupted, suggesting the existence of random Brownian motion of lipoplexes in cytosol.^140^^,^^141^ Cardarelli et al.^142^ demonstrated that, contrary to the DOTAP-based lipoplexes, Lipofectamine mostly adopted random Brownian diffusion to acquire optimal transfection. But now, there is a question of why Lipofectamine reagents are different from their counterparts (DOTAP-based lipoplexes) in the motion mode of cytoplasmic trafficking,^142^ since the diameters of particles of the former are relatively larger, typically more than 300 nm,^143^ whose movement should have been immensely restricted by the cytoskeletal network. The ability of lipoplexes to migrate within cells remains controversial and needs to be clarified. In any case, it is clear that exogenous DNA dissociated from a nanocarrier is unlikely to overcome the steric hindrance to get into the nucleus, because cells might not process the dissociative DNA in the same manner as lipoplexes.^144^ In conclusion, releasing DNA into the cytoplasm does not seem to make transfection successful, as transfection can be obtained only when cytoplasmic DNA enters into nucleus or dissociation occurs in the nucleus.^145^ Thus, the unpacking of the lipoplexes is critical for the release of the DNA therapeutic, and the location where it is released is also critical. It is best to dissociate in or around the nucleus. If the complex is disassembled far from the nucleus, it is difficult for DNA to migrate into the nucleus, and, alternatively, it may be engulfed by lysosomes again.

### Factors Affecting the Therapeutic Efficacy

#### Lipid Composition

Cationic lipids are usually composed of a hydrophobic lipid anchor group, a linker arm, and a positively charged head group,^9^ forming the unique structure of a cationic lipid. The molecular structures of cationic lipids are crucial to cell uptake, endosomal escape, and cytotoxicity.^18^^,^^146^, ^147^, ^148^, ^149^ Kolašinac et al.^95^ reported that an inverted conical shape and an aromatic molecule in cationic lipid are indispensable to the fusion induction. Bruininks et al.^16^ elucidated the molecular effects of lipid tail saturation on lipid fusion and TE and suggested that cationic lipid tail saturation is a necessary condition for these processes. Additionally, the type of cationic lipid of lipoplexes greatly affects their biodistribution and therapeutic efficacy in the body after intravenous administration.^16^ Hattori et al.^9^ reported that the cationic lipids with a different amine head group, linker arm, and the length of alkyl chains strongly influenced siRNA biodistribution after injection of cationic lipoplexes.

Fusogenic lipid, DOPE, is a neutral helper lipid that is commonly added to cationic liposomal formulation for promoting membrane fusion-mediated endosomal escape.^95^^,^^150^, ^151^, ^152^, ^153^, ^154^ Meanwhile, as mentioned above, lipoplexes could also enter into the cells via membrane fusion-mediated internalization, which favors the phosphoethanolamine (PE) lipid-enriched lipoplexes.^95^ However, the amount of DOPE added to the liposome is controversial and most likely related to the structure and σ_M_ of the cationic lipids.^2^^,^^115^ For instance, one study showed that DOPE-dominated lipoplexes (e.g., DOTAP/DOPE molar ratios at 1:3) existed in a hexagonal phase completely that would destabilize lipoplexes and easily release their cargo before uptake,^115^ which was different from the transfection results shown in another study in which the optimum formulations for CLs containing lysine-derived cationic lipid preferred 80% of DOPE content.^2^

However, despite its excellent performance in *in vitro* studies, DOPE is unsuitable for improving gene delivery *in vivo*, whereas cholesterol-rich CLs work better than their DOPE counterpart to deliver genes in the body with prolonged blood circulation time.^148^ Similar to DOPE, cholesterol-rich lipoplexes could partially induce fusion-driven cellular uptake and facilitate endosomal destabilization.^99^ Cholesterol has a much smaller hydration repulsion layer than does 1,2-dioleoyl-*sn*-glycero-3-phosphocholine (DOPC), and thus the substitution of DOPC by cholesterol facilitates the fusion of CLs with the endosomal/lysosomal membrane, resulting in strong enhancement of TE.^155^

Experiments by Hattori et al.^9^ found that cationic lipids with longer alkyl chain length or added cholesterol to liposomal formulation could stabilize the structure of lipoplexes in blood circulation and hence enhance accumulation in the lungs. However, the addition of cholesterol did not always yield consistent results for TE in a variety of lipoplexes, as the work by Hattori et al.^156^ showed that the positive impact of cholesterol on TE only occurred in CLs containing cationic lipids with short and/or unsaturated alkyl chains. Another similar study by Abumanhal-Masarweh et al.^157^ investigated the effects of acyl chain length and saturation and the addition of cholesterol on cellular uptake in 4T1 cells. They found that liposomes with longer acyl chains and monounsaturated phospholipid performed much better than did shorter tails (i.e., 18:0 > 16:0 > 14:0) and saturated phospholipid (i.e., 18:1 > 18:0) on cellular uptake. Moreover, the effects of cholesterol on cellular uptake in 1,2-dimyristoyl-*sn*-glycero-3-phosphocholine (DMPC, 14:0, melting temperature [Tm] = 24°C) and hydrogenated soybean phosphatidylcholine (HSPC, 18:0, Tm = 52°C) were quite different. The addition of cholesterol transformed the lipid bilayer of DMPC from a liquid-disordered phase to a liquid-ordered phase that facilitated cellular endocytosis, while in HSPC liposomes, a solid-ordered phase and the most favorable phase for cellular uptake, transformed to a liquid-ordered phase, which weakened the cellular uptake. It is well known that cholesterol is added to make the membrane hardened and structurally stable; however, its impact on cellular uptake is lipid-dependent, and researchers should take the Tm of lipids into account.

#### Size and Surface Properties of CLs

Lipid composition and complexation with pDNA could influence the size of CLs and zeta potential and hence biodistribution and cellular entry.^158^, ^159^, ^160^ Particles smaller than 5 or 50 nm are easily excreted by urine or lodged in the liver, respectively.^90^ However, particles larger than 50 nm are difficult to penetrate deep into the tumor and thus reduce cellular uptake.^90^ Therefore, the size-turnable delivery system has a broad application prospect in cancer treatment. Although the size-dependent internalization mechanism prevails,^70^^,^^90^ the effect of particle size on the mechanism of cellular uptake has not been clearly elucidated, which varies with cell types and lipid compositions.^115^ Nevertheless, the correlation between size and TE is still evident.^28^ Muripiti et al.^161^ disclosed that TE varied with the change of charge ratios, and charge ratios at 4:1 obtained the highest TE due to their least size of ~200 nm and optimal zeta potential. Sakai-Kato et al.^162^ found that in a certain range (100–200 nm), the TE of lipoplexes was enhanced with the increase of particle size, which was due to the strong electrostatic interaction between CL and plasma membrane. Instead, however, Bruininks et al.^16^ found that fusion efficiency decreased as the size of lipoplexes increased because larger lipoplexes were more stable than their smaller counterparts during the process of endosomal membrane fusion.

Besides size, surface charge of the liposome can also affect its behavior in the body.^163^ Permanently charged cationic lipids are not conducive to good pharmacokinetics. Specifically, excessive positive charges are easily recognized by the immune system for accelerated removal, and aggregation occurs when electrostatic repulsion is too low, leading to accumulation in “first pass” organs.^46^ Unlike the uncertainty of the influence of particle size on TE, the impact of particle σ_M_ on TE is obvious. High σ_M_ of lipoplexes increases the structural stability of lipid/DNA complexes and the electrostatic interaction with biological membranes, but at the same time, their cytotoxicity and difficulty of intracellular complex dissociation also increase.^128^ The addition of PEG-lipid and neutral helper lipid into CLs alters their σ_M_,^164^ resulting in a relative stable structure and PC reduction that extend blood circulation time of CLs,^60^^,^^165^ but poor adhesion to vessel walls and tumor penetration.^166^^,^^167^

Nowadays, it is a trend for ligand-receptor targeting in nanocarriers, because many specific receptors are more highly expressed in tumor than in normal tissues.^70^^,^^168^ Targeting ligands such as folate,^169^, ^170^, ^171^, ^172^ transferrin,^173^^,^^174^ and the cyclic Arg-Gly-Asp (cRGD) peptides^175^^,^^176^ are exploited to enhance tumor-targeted delivery, making it more effectively and rapidly internalized via receptor-mediated endocytosis of target cells. In recent years, dual-targeted nanomedicines are expected to overcome the uncontrollable and fluctuating targeting efficiency of single-ligand targeting, resulting in better cell selectivity and gene therapy.^177^^,^^178^ These surface modifications can work in different parts, such as plasma membrane,^173^^,^^179^ endosomal compartment,^27^^,^^180^ or even the nuclear envelope,^181^^,^^182^ making CLs versatile.

### Conclusions

CL is a promising nanocarrier for gene delivery thanks to its large-scale preparation, biodegradability, and biocompatibility. The positive charge of CLs contributes to their interaction with cells and endosomal escape, but also inevitably increases the immunogenicity and toxicity of CLs in the body, leading to rapid clearance by the RES. In contrast, Onpattro (patisiran), the US Food and Drug Administration (FDA) first approved RNAi drug, used lipid nanoparticles as delivery vectors, suggesting that low zeta potential is a necessary condition for obtaining good pharmacokinetics.^183^ The successful clinical translation of Onpattro depends on its ionizable property (i.e., nearly neutral in blood but positively charged in endosomal compartment) and defined size (50 nm)^184^ by incorporating PEG-lipids, which indicates that the positive charge of CLs should be compromised between high TE and good pharmacokinetics. The relative traditional CLs, which have been favored by many researchers, are now greatly challenged. They should ensure high TE and at the same time obtain good pharmacokinetic properties to meet the clinical needs. Therefore, the clinical success of lipoplex-based cancer therapy requires designers to pay close attention to the barriers of lipoplexes in gene delivery and the impact of physicochemical properties of CLs on the therapeutic outcome.

## Author Contributions

C.L. conducted the writing and literature review of the entire article; L.Z. and N.D. conducted the modification and proofreading of the entire article; and W.Z., X.C., R.G., and H.S. conducted the conception of the whole review and the final proofreading.

## Conflicts of Interest

The authors declare no competing interests.
